# Supplementary material for: Evolutionary dynamics and genomic features of the Elizabethkingia anophelis 2015 to 2016 Wisconsin outbreak strain
Source: Nat Commun. 2017 May 24;8:15483. doi: 10.1038/ncomms15483 (PMC5458099; doi:10.1038/ncomms15483)
Supplement: Supplementary Information — Supplementary Figures, Supplementary Tables, Supplementary Methods and Supplementary References. [file ncomms15483-s10.pdf]

1. Supplementary Figures

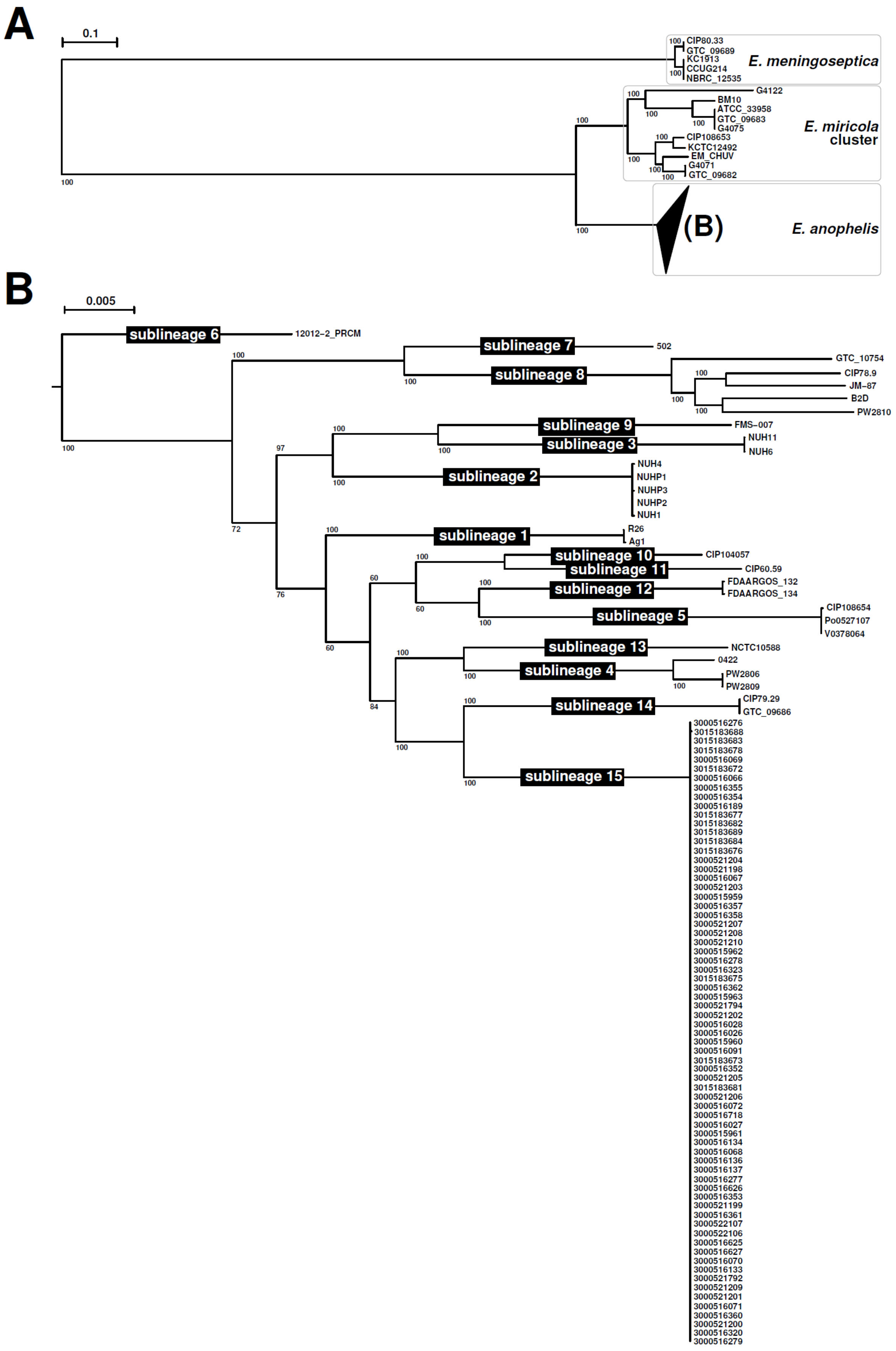

Supplementary Fig. 1. Phylogenetic tree of *Elizabethkingia* strains and position of the outbreak strain within *E. anophelis*

Maximum likelihood phylogenetic tree of 114 *Elizabethkingia* isolates inferred from 1,662,672 aligned nucleotide characters (554,224 codons). Confidence support at branches corresponds to ultrafast bootstrap values as implemented in IQ-TREE (1,000 replicates) [1]. Scale bars indicate the average number of substitutions per site. (A). Phylogenetic tree for the genus. The *E. anophelis* clade is represented by a black triangle denoted B, angle depth positions being proportional to the observed minimum (lower angle) and maximum (upper angle) root-to-tip distances within the inferred subtree. Note that the *E. miricola* cluster is heterogeneous and may contain several species (B). Detailed *E. anophelis* subtree. Sublineage numbers are indicated inside black boxes placed on their corresponding branches.

DOI: https://doi.org/10.6084/m9.figshare.4585492.v1

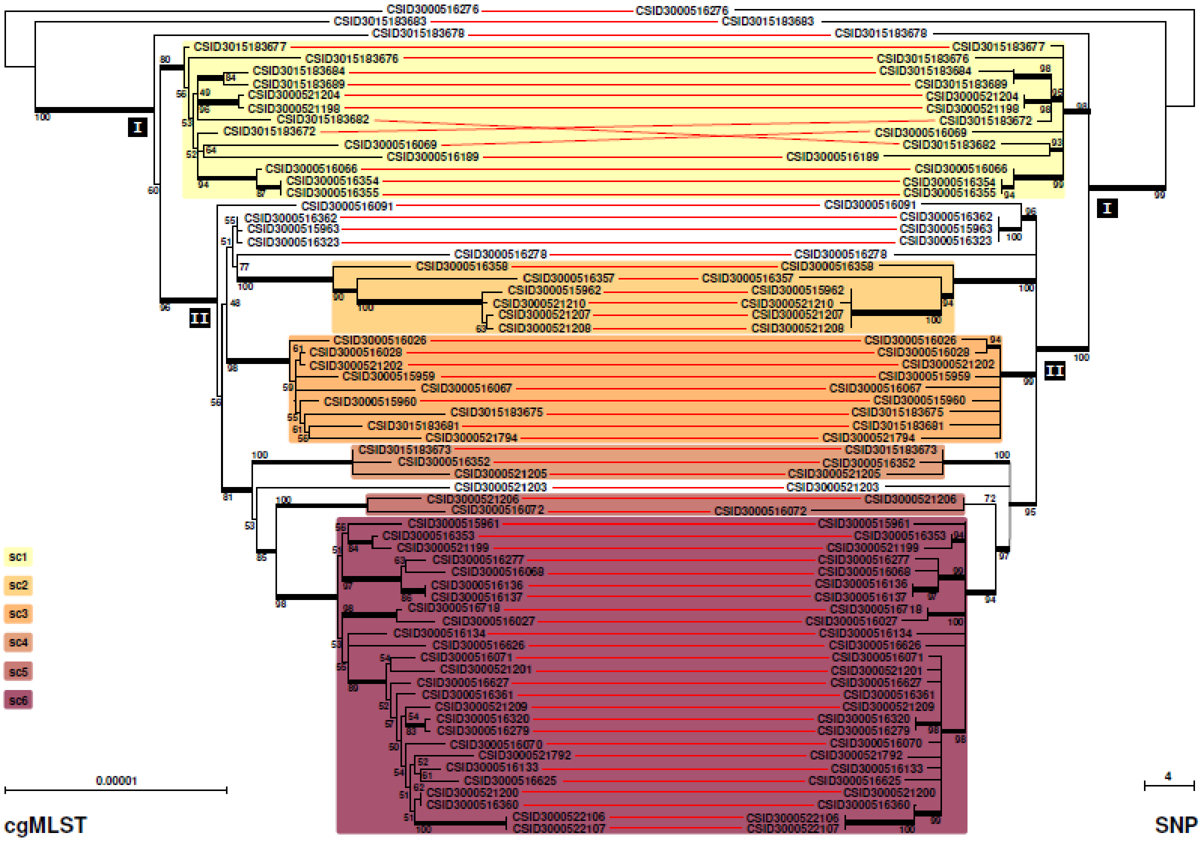

Supplementary Fig. 2. Phylogenetic trees inferred from cgMLST and SNP data. The cgMLST tree (left) is identical to the one in Figure 1 (without the *mutS* isolate CSID 3015183688). The SNP tree (right) was inferred from SNP data following the same phylogenetic method as for cgMLST (see Methods) but based on the nucleotide evolutionary model TVM [2]. Positions of each isolate in both trees are joined by a red line. Sub-clusters (sc) 1 to 6 are represented by coloured boxes as in Figure 1.

DOI: https://doi.org/10.6084/m9.figshare.4585495.v2

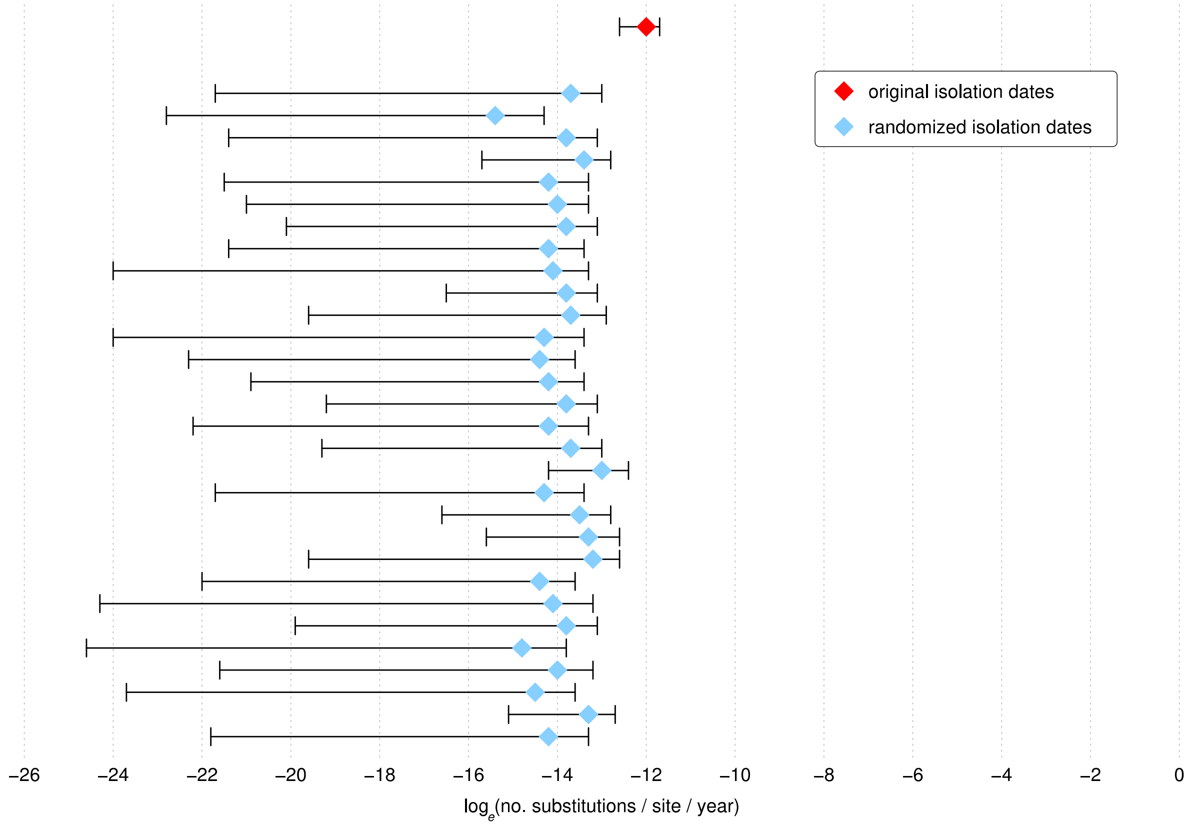

Supplementary Fig. 3. BEAST randomization test for temporal signal. Natural logarithm of the posterior mean (diamonds) and 95% highest posterior densities (bars) of the substitution rate estimated from the outbreak data (upper, red) and 30 tip-date randomizations (bottom, light blue).

DOI: https://doi.org/10.6084/m9.figshare.4593325.v1

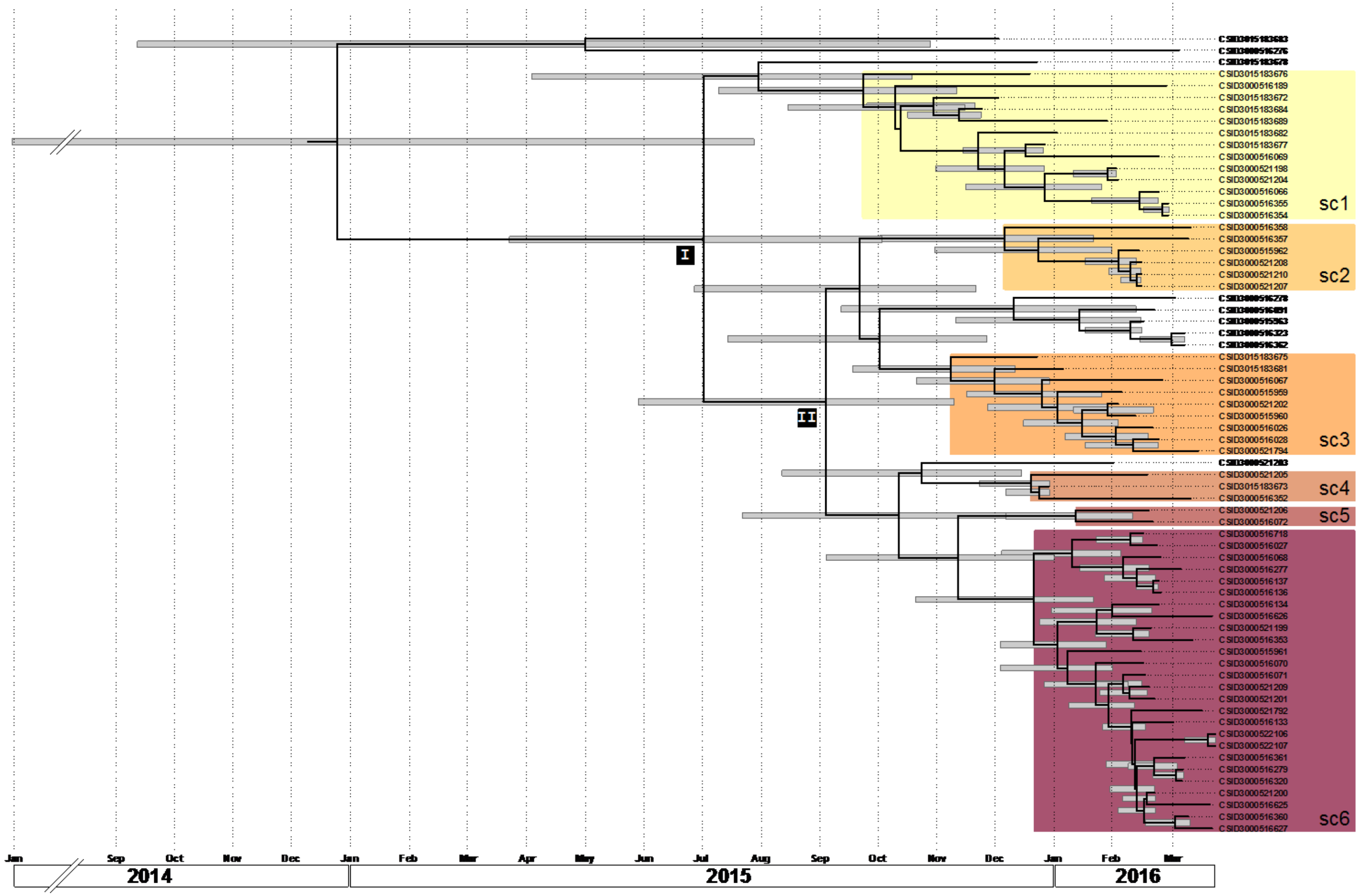

Supplementary Fig. 4. Bayesian maximum-clade-credibility time-scaled phylogenetic tree of the outbreak isolates. Sub-clusters (sc) 1 to 6 are represented by coloured boxes. Time is indicated on the x-axis; vertical dashed lines correspond to the first day of the indicated month. Light grey horizontal bars centered on nodes represent 95% HPD for the estimated node age.

DOI: https://doi.org/10.6084/m9.figshare.4593328.v1

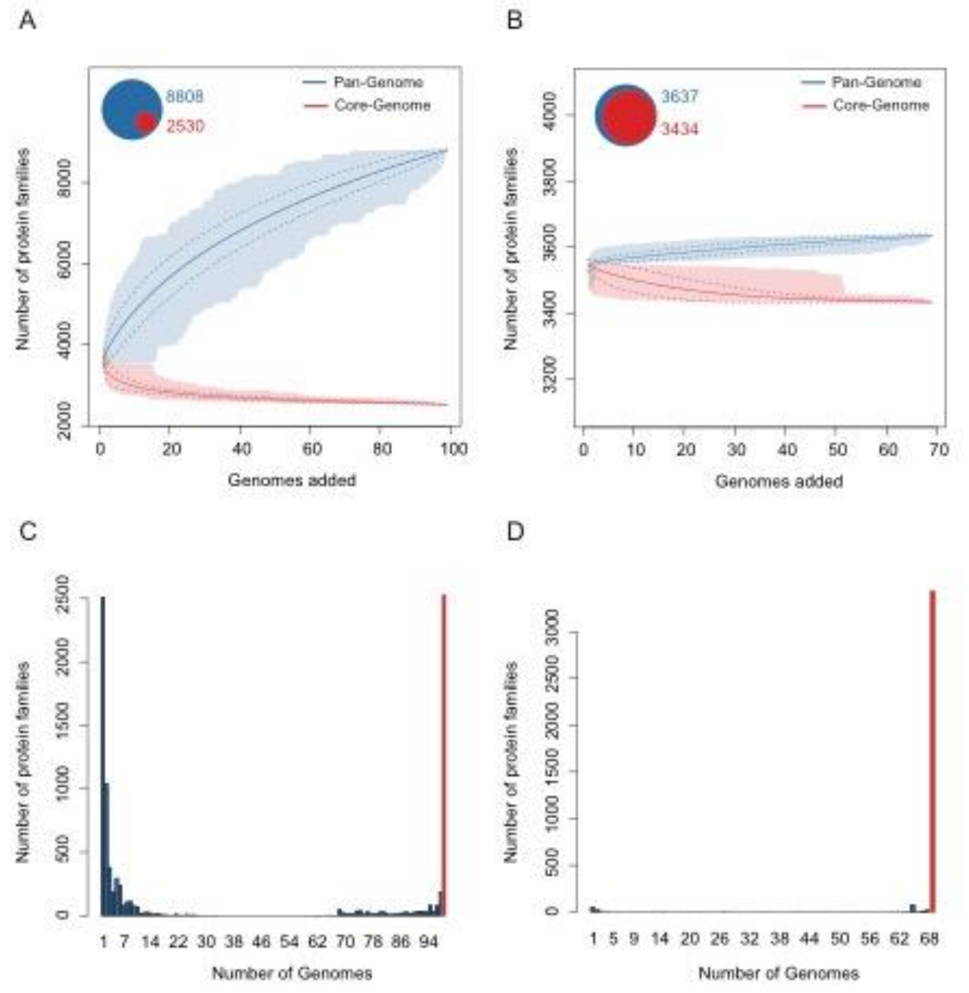

Supplementary Fig. 5. Core- and pan-genomes of *E. anophelis* and distribution of protein families. Data are shown for (A, C) all 99 sequenced *E. anophelis* genomes and (B, D) 69 sequenced *E. anophelis* genomes from the Wisconsin outbreak. (A) and (B) show the core- and pan-genome accumulation curves calculated using the statistical software R (R Core Team 2014). These curves describe the change in total number of protein families (pan-genome; in blue) and protein families in common (core-genome; in red) obtained as more genomes are included in the sample. The procedure was repeated 1,000 times by randomly modifying the order of integration of genomes in the analysis. Solid lines correspond to the average number of protein families obtained across all permutations, dashed lines indicate standard deviation of the mean, and shaded regions indicate range. (C) and (D) show the distribution of protein families, i.e. the number of protein families of the pan-genome found in the number of genomes indicated on the X axis: from 1 for strain-specific proteins, to 99 for *E. anophelis* core-protein families.

DOI: https://doi.org/10.6084/m9.figshare.4593343.v2

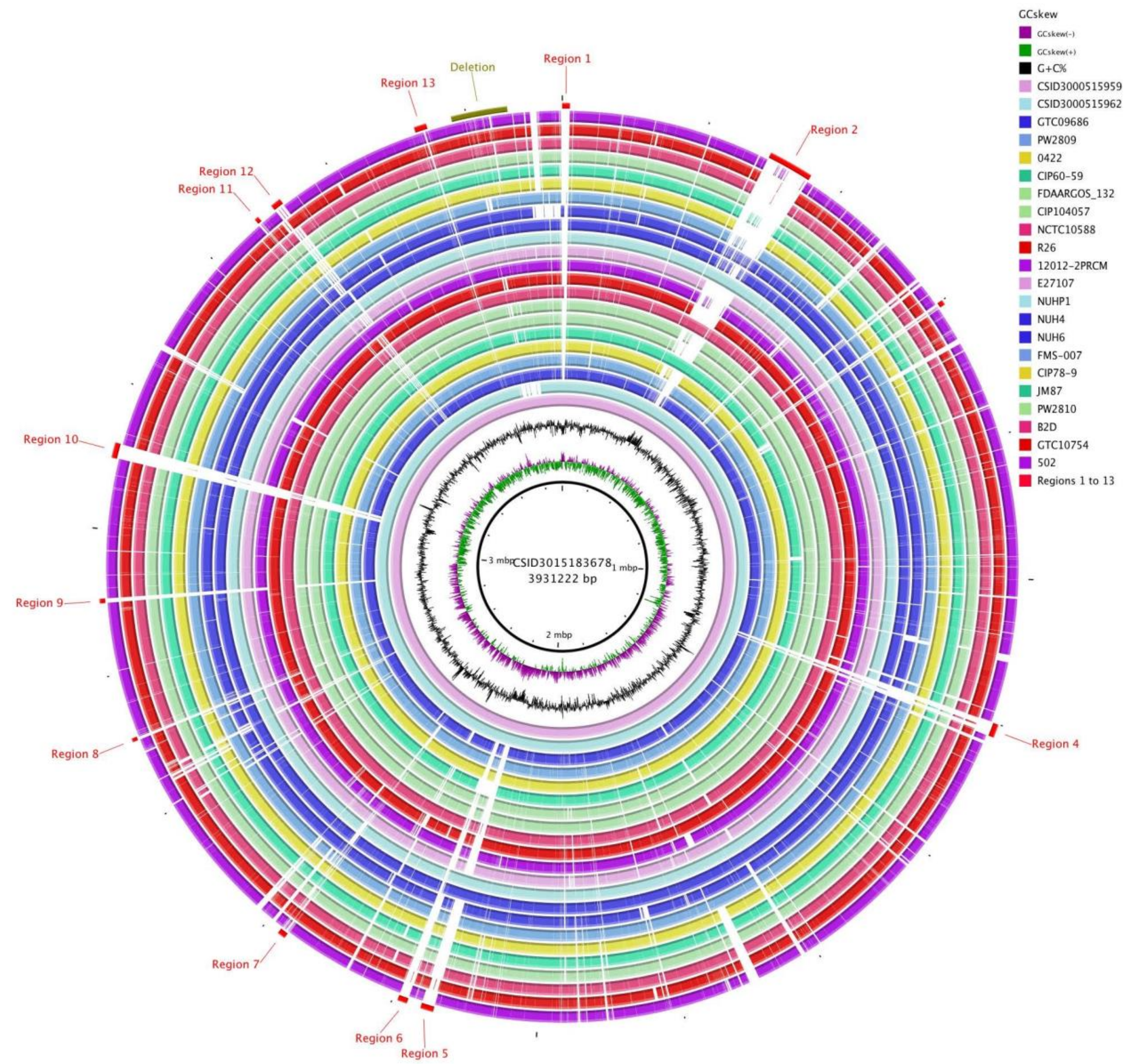

Supplementary Fig. 6. Circular representation of gene conservation between the reference outbreak isolate *E. anophelis* CSID 3015183678 and other *E. anophelis* genomes. Circles are numbered from 1 (innermost circle) to 26 (outermost circle). Circle 1: scale of the CSID 3015183678 genome. Circle 2: GC skew (positive GC skew, green; negative GC skew, violet). Circle 3: G+C content (above average, external peaks; below average, internal peaks). Circle 4: all Wisconsin outbreak isolates except the 4 isolates in circle 5. Circle 5: Wisconsin outbreak isolates CSID 3000515962, CSID 3000521207, CSID 3000521208, CSID 3000521210, which are deleted for a 77 kb region (Deletion, last circle). Circle 6: strain GTC09686. Circle 7: strain PW2809. Circle 8: strain 0422. Circle 9: strain CIP60.59. Circle 10: strain FDAARGOS_132. Circle 11: strain CIP104057. Circle 12:

strain NCTC10588. Circle 13: strain R26. Circle 14: strain 12012-2PRCM. Circle 15: strain E27107. Circle 16: strain NUHP1. Circle 17: strain NUH4. Circle 18: strain NUH6. Circle 19: strain FMS-007. Circle 20: strain CIP78.9. Circle 21: strain JM87. Circle 22: strain PW2810. Circle 23: strain B2D. Circle 24: strain GTC10754. Circle 25: strain 502. Circle 26: outbreak-associated genomic regions 1 to 13, and deletion. This representation was performed using BRIG with options: “blastn -F F –e 0.001 –W 10”.

DOI: https://doi.org/10.6084/m9.figshare.4593346.v2

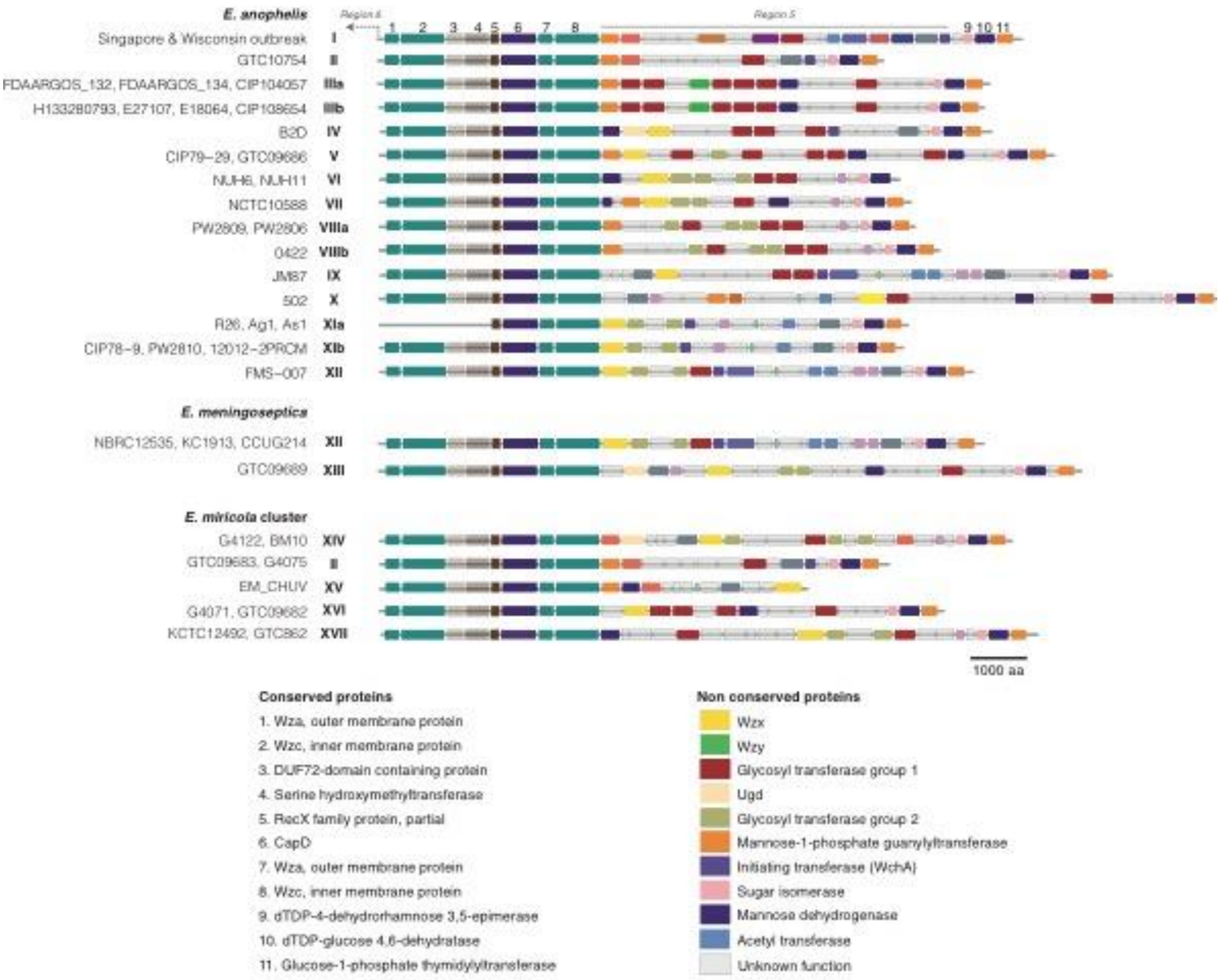

Supplementary Fig. 7. Genetic organization of the putative capsular polysaccharide synthesis (*cps*) protein clusters in *Elizabethkingia* genomes. Open reading frames (ORFs) are shown as rectangles and are drawn to scale. Intergenic spaces are not to scale. Numbers on top of the first genome correspond to the conserved proteins and their annotation is detailed at the bottom of the figure. Some of the *cps* proteins typically associated to Wzy-dependent capsules, namely Wzx flippase (yellow) and Wzy polymerase (green), could not be confidently identified in all serotypes due to their poor conservation even among phylogenetically closely-related genomes. Other common polysaccharidic enzymes associated to capsule synthesis are also indicated. Variants are described for several putative serotypes (IIIa/b, VIIIa/b and XIa/b), on the basis that the small genomic discrepancies observed are not expected to result in major changes in sugar composition. The scale represents 1,000 amino acids. The star (*) indicates *E. meningoseptica* genomes that were replicates of the type strain genome from various culture collections.

DOI: https://doi.org/10.6084/m9.figshare.4593349.v2

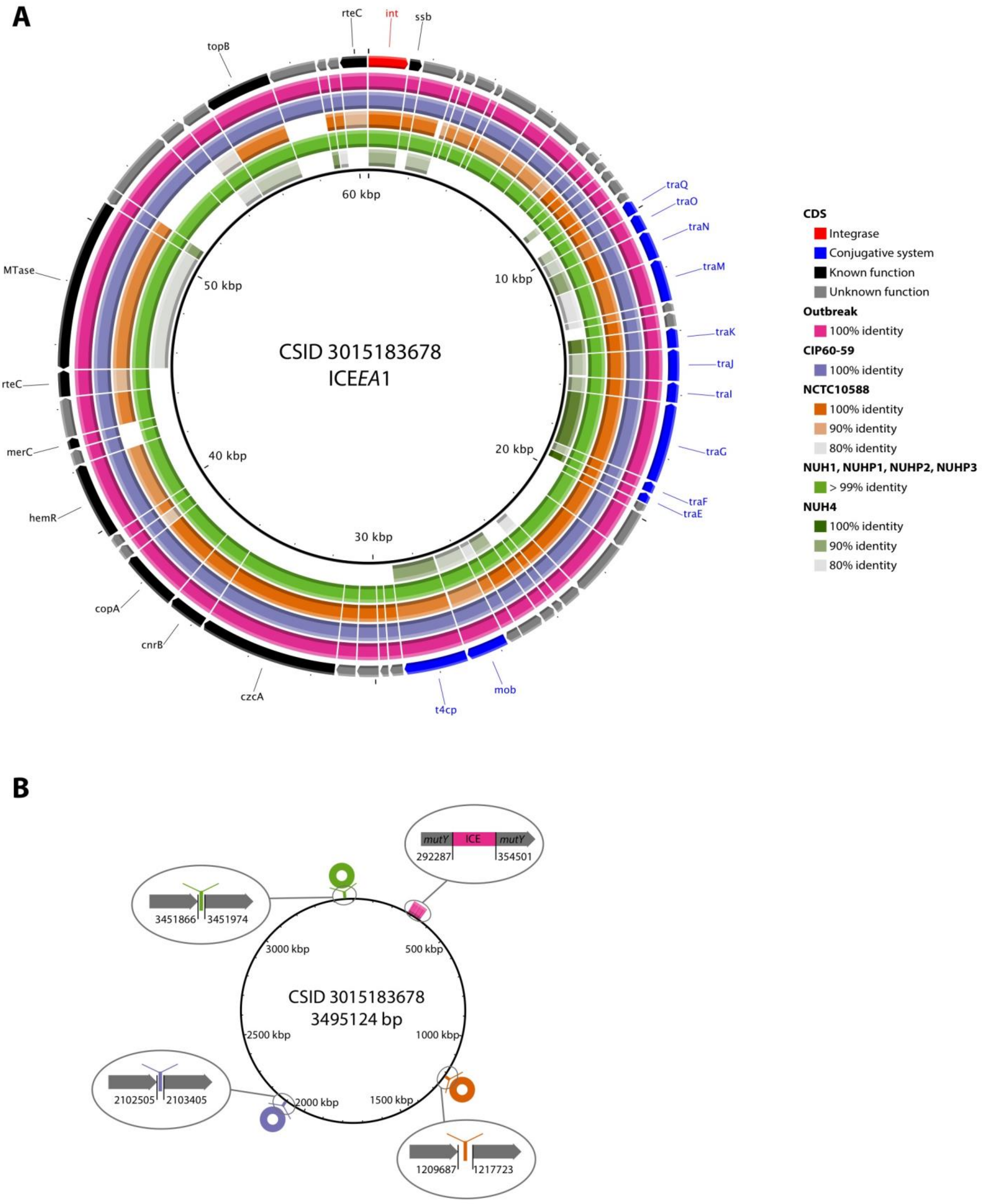

Supplementary Fig. 8. Circular representation of the integrative and conjugative element ICE*Ea*1 and gene conservation and ICE localization among representative *E. anophelis* genomes

(A) ICE*Ea*1 content among representative genomes. Circles are numbered from 1 (innermost circle) to 6 (outermost circle). The protein coding genes of ICE*Ea*1 in the reference genome CSID

3015183678 (Circle 6) are compared to: Circle 1: Singapore outbreak strain NUH4; Circle 2: Singapore outbreak strains NUH1, NUHP1, NUHP2 and NUHP3 (all identical). Circle 3: strain NCTC 10588; Circle 4: strain CIP60.59; Circle 5: the 68 other Wisconsin outbreak isolates. On circle 6, genes of the conjugative system are colored in blue, those with a known function in black, and those with unknown function in gray. The figure was obtained using BRIG with option: “blastp –evalue 0.001 –seg no”, and representing, for each genome, only the proteins resulting from bidirectional best hit (BBH) with 80% similarity or more, and showing synteny (at least 4 syntenic proteins among a radius of 5) with the reference ICE proteins. Identity percentage in the legend is given for deduced amino acid sequences. (B) Relative position on the reference genome of the ICE*Ea*1 element in all the strains that harbor it (same color code as in (A)).

DOI: https://doi.org/10.6084/m9.figshare.4593352.v2

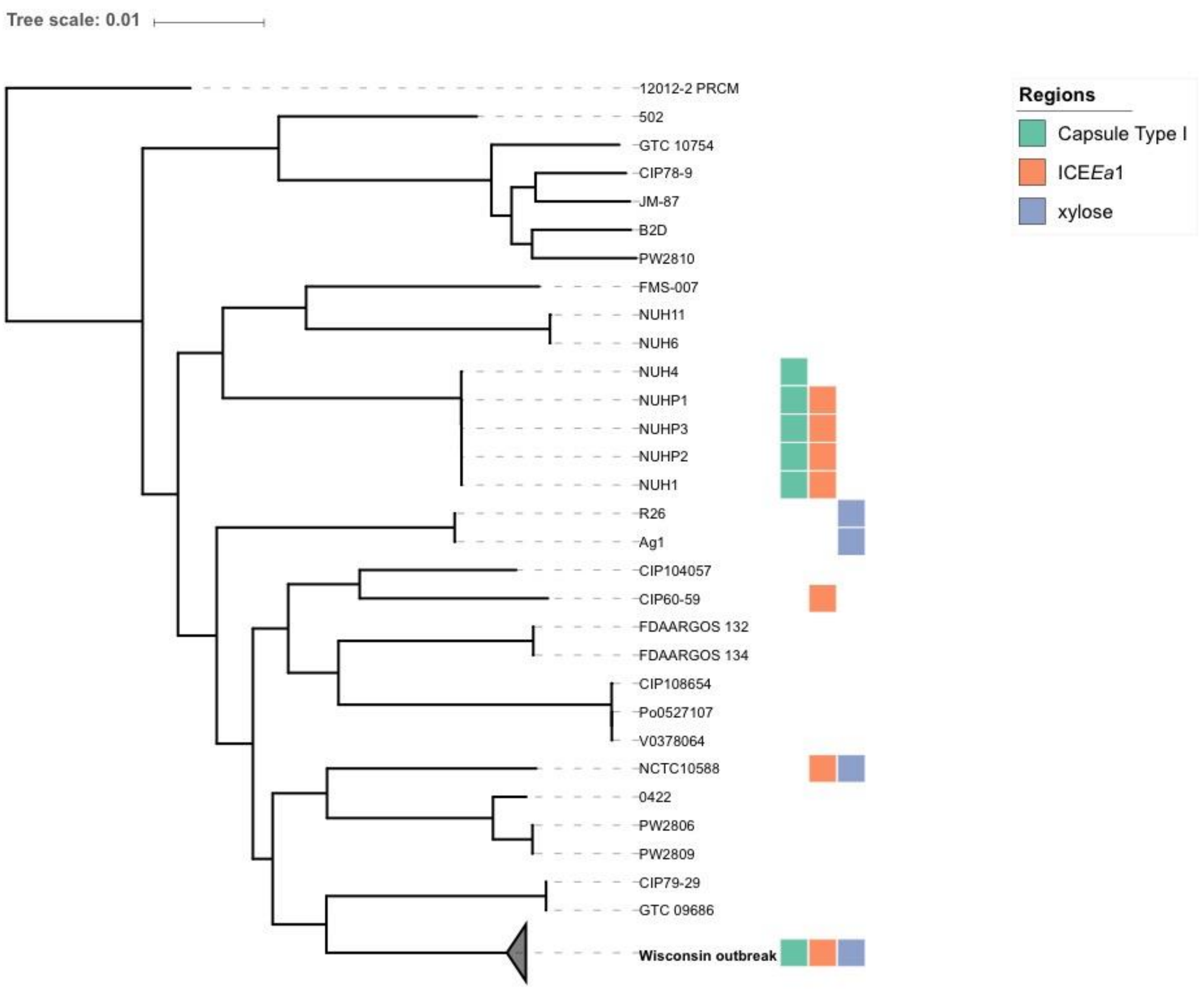

Supplementary Fig. 9. Phylogenetic distribution of ICE*Ea*1, the xylose cluster and the Type I *cps* cluster.

The phylogeny is the same as in Supplementary Figure 1 B, except that all outbreak isolates are collapsed into a grey triangle because they have the same features depicted here. Coloured squares are added after each strain name having the following features: a capsule of Type I (green square), the ICE*Ea*1 element (orange square), or the xylose cluster (violet square). The figure was obtained with the help of iTOL [3].

DOI: https://doi.org/10.6084/m9.figshare.4593355.v2

2. Supplementary Tables

Supplementary Table 1: General characteristics of core- and pan-genomes

**Dataset**	**Number of Genomes**	**Average Proteome***	**Total number of proteins**	**Core-Genome***	**Pan-Genome**
E. anophelis (outbreak)	69	3545	244605	3434	3637
E. anophelis (all)	99	3589	355311	2530	8808

(*) inferred from the pan-genome

3 Supplementary methods

3.1 Suspicion of *mutS* mutation leading to hypermutator phenotype, and measurement of the *in vitro* mutation frequency of the *mutS* mutant and other outbreak isolates

Visual inspection of the complete circularized genome alignment of all outbreak strains revealed that the genome of isolate CSID 3015183688 had 21 instances of the addition or deletion of a single nucleotide, primarily within homopolymeric runs of nucleotides, leading us to suspect a defect in its DNA repair machinery. This hypermutator hypothesis was supported by the discovery of a single nucleotide substitution in the methyl-directed mismatch repair *mutS* gene of isolate CSID 3015183688. The mutation causes the MutS protein to be truncated for the 29 C-terminal amino-acids. The C-terminal portion of the MutS protein is necessary for oligomerization, and deletion of as few as 7 C-terminal amino acids was shown to disrupt its function in *E. coli* [4]. We therefore surmised that this truncation would produce a strong mutator phenotype in *Elizabethkingia* as well.

To confirm this and estimate the mutation frequency of other outbreak isolates, a time series experiment was performed over the course of 44 days. Accumulation of SNPs was measured in real time for 3 of the outbreak isolates. Isolates were streaked from frozen stock onto heart infusion agar supplemented with 5% rabbit blood agar and grown at 35°C. Then, a single colony was subcultured each day. Every third day until day 19 and every 7th day from day 17 until day 44, DNA was extracted, and complete circularized genomes were generated, assembled, and aligned for SNP evaluation, as described in the main Methods section.

We found that two SNPs appeared in each of the control isolates CSID 3015183673 (between day 0 and day 1, and between day 23 and 30) and CSID 3015183678 (between day 13 and day 16, and between day 30 and 37), whereas the putative hypermutator isolate CSID 3015183688 generated between 2 and 8 SNPs (average = 4.1 SNPs) over each 3-day interval, and between 10 and 24 SNPs (average = 15.5) over each 7-day interval, resulting in a total of 85 SNPs over the 44 days.

3.2 Phylogenetic analysis using SNPs

Phylogenetic analysis was performed based on the final set of n=374 filtered SNPs representing substitution mutations that arose within the population of outbreak isolates (see main text). The isolate GTC_09686 (SRA accession: DRR015707) was included as an outgroup to root the tree. The concatenated alignment of these SNP alleles was used to generate a ML phylogenetic tree (**Supplementary Figure 2; Supplementary Data 1**) with a GTR model with a gamma distribution of rate heterogeneity (GTR+G) using RAxML v8 [5].

3.3 Temporal analysis using mapping-based SNPs

Temporal signal in the ML tree was initially explored using TempEst [6], excluding the *mutS* hypermutator strain and outgroup. Detailed phylodynamic modelling was then conducted using BEAST v1.8.3 [7]. For BEAST analysis, we excluded the *mutS* hypermutator strain (because this strain had a highly distinctive evolutionary rate which may confound the analyses) and, as a simplification for calculation speed, all sites that were non-variant within the remaining outbreak isolates, leaving n=290 SNPs for analysis in 68 outbreak isolates. Isolation dates were expressed in days. Preliminary runs comparing alternative substitution models (Hasegawa, Kishino and Yano model [HKY] vs. GTR + G), alternative clock models (strict vs. relaxed lognormal) and population models (constant size vs. Bayesian skyline) found support for the HKY model, rate variation and constant population size (as assessed by estimates of rate standard deviation and Bayes Factor analysis). Ten replicate chains using the HKY substitution model with relaxed lognormal clock and constant population size model were run with different random seeds for 50 million iterations. All ten chains converged, and these all yielded closely overlapping 95% HPD intervals on estimates of mean mutation rate and root height. Data from all runs were combined using LogCombiner (excluding the first 10% of each as burn-in) to yield final estimates and 95% HPDs for reported parameters, and a maximum clade credibility tree. Mutation rates estimates extracted from BEAST results in units of substitutions per site per day were converted to substitutions per genome per year by multiplying by 290 x 365 (SNP sites per alignment x days per year); and to substitutions per site per year by further dividing by 3,571,924 (total genomic sites assayed for SNPs).

4. Supplementary References

1. Minh, B. Q., Nguyen, M. A. T. & von Haeseler, A. Ultrafast approximation for phylogenetic bootstrap. *Mol. Biol. Evol.* **30,** 1188–1195 (2013).

2. Posada, D. Using MODELTEST and PAUP* to select a model of nucleotide substitution. *Curr. Protoc. Bioinforma.* **Chapter 6,** Unit 6.5 (2003).

3. Letunic, I. & Bork, P. Interactive tree of life (iTOL) v3: an online tool for the display and annotation of phylogenetic and other trees. *Nucleic Acids Res.* **44,** W242–W245 (2016).

4. Miguel, V., Pezza, R. J. & Argaraña, C. E. The C-terminal region of *Escherichia coli* MutS and protein oligomerization. *Biochem. Biophys. Res. Commun.* **360,** 412–7 (2007).

5. Stamatakis, A. RAxML version 8: a tool for phylogenetic analysis and post-analysis of large phylogenies. *Bioinformatics* **30,** 1312–3 (2014).

6. Rambaut, A., Lam, T. T., Max Carvalho, L. & Pybus, O. G. Exploring the temporal structure of heterochronous sequences using TempEst (formerly Path-O-Gen). *Virus Evol.* **2,** vew007 (2016).

7. Drummond, A. J. & Rambaut, A. BEAST: Bayesian evolutionary analysis by sampling trees. *BMC Evol. Biol.* **7,** 214 (2007).
